# Microfluidic and Organ-on-a-Chip Approaches to Investigate Cellular and Microenvironmental Contributions to Cardiovascular Function and Pathology

**DOI:** 10.3389/fbioe.2021.624435

**Published:** 2021-02-04

**Authors:** Elizabeth L. Doherty, Wen Yih Aw, Anthony J. Hickey, William J. Polacheck

**Affiliations:** ^1^Joint Department of Biomedical Engineering, University of Carolina at Chapel Hill and North Carolina State University, Chapel Hill, NC, United States; ^2^University of North Carolina Catalyst for Rare Diseases, Eshelman School of Pharmacy, University of North Carolina at Chapel Hill, Chapel Hill, NC, United States; ^3^RTI International, Durham, NC, United States; ^4^Cell Biology and Physiology, University of North Carolina at Chapel Hill School of Medicine, Chapel Hill, NC, United States; ^5^McAllister Heart Institute, School of Medicine, University of North Carolina at Chapel Hill, Chapel Hill, NC, United States

**Keywords:** microfluidics, endothelial cells, cardiomyocytes, vascular biology, rare disease, microfabrication, biofluid mechanics, organ-on-chip

## Abstract

Over the past decade, advances in microfabrication and biomaterials have facilitated the development of microfluidic tissue and organ models to address challenges with conventional animal and cell culture systems. These systems have largely been developed for human disease modeling and preclinical drug development and have been increasingly used to understand cellular and molecular mechanisms, particularly in the cardiovascular system where the characteristic mechanics and architecture are difficult to recapitulate in traditional systems. Here, we review recent microfluidic approaches to model the cardiovascular system and novel insights provided by these systems. Key features of microfluidic approaches include the ability to pattern cells and extracellular matrix (ECM) at cellular length scales and the ability to use patient-derived cells. We focus the review on approaches that have leveraged these features to explore the relationship between genetic mutations and the microenvironment in cardiovascular disease progression. Additionally, we discuss limitations and benefits of the various approaches, and conclude by considering the role further advances in microfabrication technology and biochemistry techniques play in establishing microfluidic cardiovascular disease models as central tools for understanding biological mechanisms and for developing interventional strategies.

## Introduction

Cardiovascular disease is the leading cause of death among Americans (Heron and Anderson, 2016), and vascular dysfunction is not only a hallmark but often the underlying cause of morbidity and mortality in many diseases (Rajendran et al., 2013). Current approaches for drug development to treat cardiovascular diseases rely primarily on animal models that lack complete human physiology and do not recapitulate the full disease phenotype. In addition, many inherited diseases are associated with multiple genetic mutations thus requiring multiple animal models for a single disease. Furthermore, studies of disposition and efficacy of drugs in animals or *ex-vivo* human tissue do not allow a scale of scrutiny that informs our understanding of events in the microenvironment that often characterize genetic disorders or are the foundation for more serious systemic disease. These limitations introduce challenges for inferring therapeutic efficacy in patients based on interventions in animal models, particularly for diseases in which the genetics are not well-characterized, such as rare inherited diseases. An alternative to animal models and organotypic culture are conventional *in-vitro* models, which are commonly used in pharmacokinetic studies and preclinical drug discovery. 2D cell culture models offer simpler, more inexpensive, and higher throughput platforms for discovery and screening. While traditional cell culture systems allow genetic manipulations and offer compatibility with biochemical assays that are not possible in animal models, cells cultured on stiff surfaces do not experience mechanical stimuli and topography of tissue microenvironment and are less reflective of the *in-vivo* conditions.

Microfluidic technology allows fabrication of systems populated by human cells that recapitulate some aspects of organ function (Huh et al., 2010; Bhatia and Ingber, 2014; Low et al., 2020) and can be used to probe key molecular or cellular events, in a physiologically relevant construct, to explore the origins of acute or chronic disease. These microfluidic “organ-on-chip” models have particular relevance in the study of cardiovascular disease where functional outputs such as resistance to mass transport, fluid displacement, and mechanical force can be well-characterized. A key feature of the most recent of such models is the control over microenvironmental parameters such as the content and mechanical properties of extracellular matrix, the inclusion of stromal and supporting cell types, recapitulation of tissue architecture, and physiologic hemodynamics (Nam et al., 2015; Bordeleau et al., 2017; Partyka et al., 2017; Kaneko et al., 2018; Polacheck et al., 2019; Atchison et al., 2020). When combined with modern cellular and molecular techniques, including induced pluripotent stem cell (iPSC) technology, novel methods for isolation and maintenance of primary cells, and gene editing tools, microfluidic cardiovascular models provide a powerful platform for isolating factors associated with cardiovascular disease.

**Figure 1:**
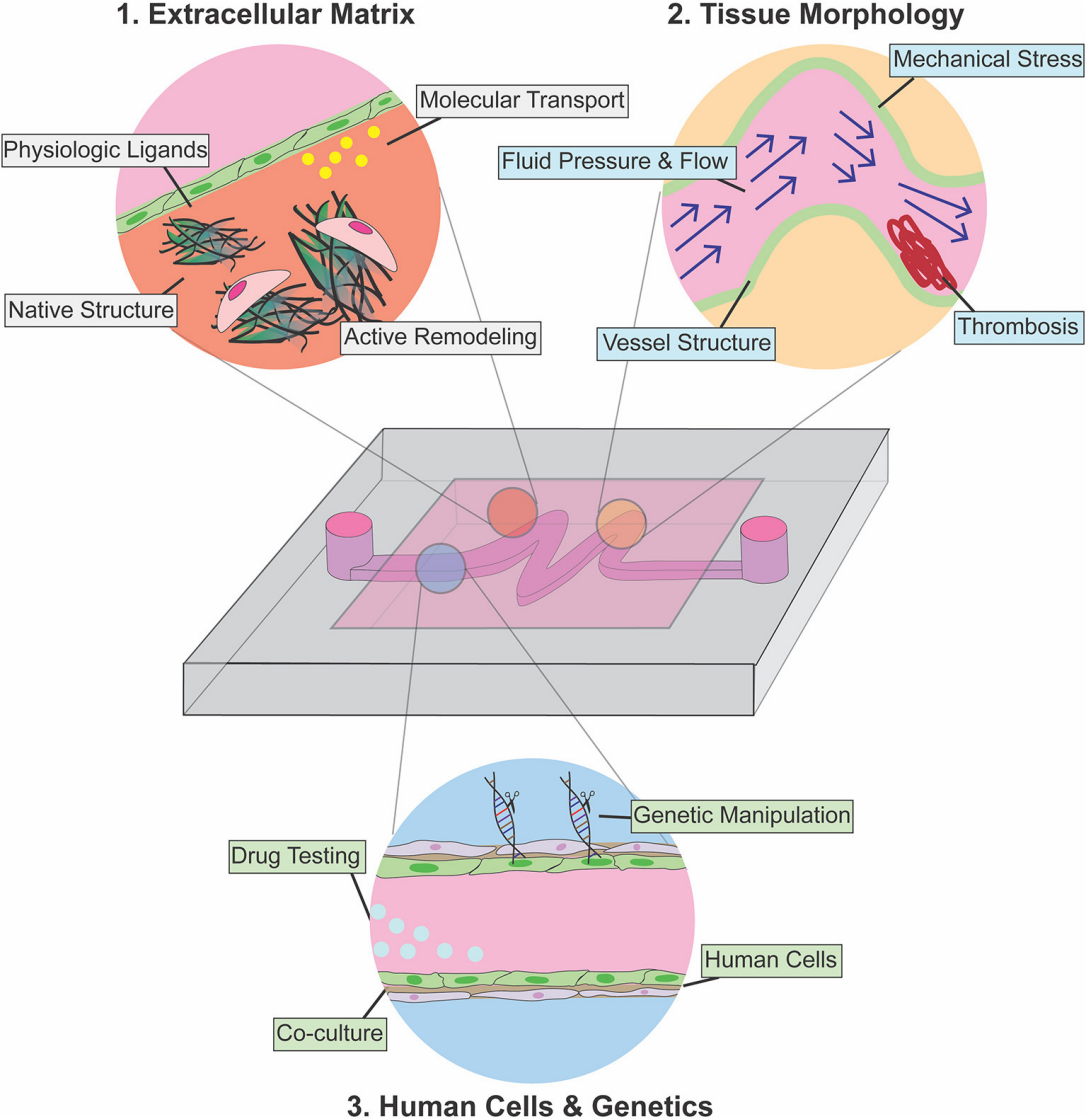
Use of microfluidic devices for modeling cardiovascular disease pathophysiology. Introduction of physiologic ECM enables the investigation of cellular remodeling of ECM in disease conditions (red), and microfluidics also allows patterning and recapitulation of altered tissue morphology and abnormal hemodynamics associated with cardiovascular diseases (yellow). Integration of gene editing with microfluidic technology allows modeling of endothelium-stromal cells interactions within physiological microenvironment in normal development and disease condition (light blue).

Here, we review microfluidic models that have been developed to study cardiovascular function and pathology from a genetic and microenvironmental perspective. We focus on platforms that have elucidated the role of physical forces in pathophysiology, and we highlight examples of devices that have been used as a testbed for therapeutic intervention. We divide the review into three separate but interrelated sections that elaborate on key parameters of cardiovascular tissue architecture that can be separately controlled and analyzed when using microfluidic approaches as compared to traditional *in-vitro* or animal models (Figure 1): (1) Extracellular matrix (ECM)—here we discuss examples of devices developed to recapitulate and pattern native ECM *ex-vivo*, with a focus on models developed to understand the role of ECM in disease progression; (2) Tissue Morphology—we then review devices that leverage the ability to pattern fluid, polymers, and cells to allow modeling of patient-specific pathophysiologic vascular architecture to investigate how tissue structure contributes to disease progression; (3) Cellular Genetics—in the final section, we discuss devices that incorporate primary patient cells and human cells engineered to express disease-specific mutations to characterize the effects of these mutations on tissue function. Combining knowledge and approaches from each of these focus areas is essential to future developments. We conclude with a perspective on how advances in genetic screening and manipulation could further be integrated with next-generation microfluidic models to provide mechanistic insight and screening for interventional strategies for rare cardiovascular diseases for which animal and *ex-vivo* tissue models are unsuitable.

## Extracellular Matrix

The cardiovascular system is a closed, pressurized system, which utilizes cyclic pumping to enable convective transport of oxygen and nutrients throughout the body. Thus, the cells and extracellular matrix (ECM) that comprise cardiac and vascular tissue must withstand cyclic pressures and hemodynamic fluid stresses of magnitudes that vary with location within the circulatory system (Secomb, 2016). As a result, the structure and composition of ECM within the cardiovascular system is highly complex and tissue-specific (Potente and Mäkinen, 2017). For example, while vessel ECM is generally composed of fibrillar collagens, elastic fibers, fibronectin, fibrillin, and proteoglycans, the ratio of collagens and elastic fibers vary across vessels. Collagens function to provide tensile strength, and together with elastic fibers ensure vessels elasticity (Wagenseil and Mecham, 2009; Cheng and Wagenseil, 2012), and thus proximal arteries that experience higher wall tension have a higher number of collagens and elastin than distal arteries and veins (Clark and Glagov, 1985). Furthermore, changes in matrix composition and structure are intimately linked with cardiovascular development and disease, and we direct the reader to comprehensive reviews that catalog key ECM constituents in myocardial and vascular ECM (Davis and Senger, 2005; Wagenseil and Mecham, 2009; Rienks et al., 2014). In addition to its structural role, the ECM presents biochemical and biophysical cues to adherent cells that regulate diverse processes from cell fate (Frantz et al., 2010; Cox and Erler, 2011; Daley and Yamada, 2013) to proliferation and apoptosis (Chen et al., 1997). For example, ECM composition modulates developmental angiogenesis through integrin-dependent signaling (Hynes, 2007) and proteoglycan content in the ECM is known to play a critical role in cardiac development and cardiomyocyte differentiation (Chan et al., 2010). Given the central importance of the ECM in cardiovascular development, homeostasis, and disease pathogenesis, the development of cardiovascular disease models requires careful consideration of the composition and architecture of the ECM. Here we review recent progress in the integration of ECM scaffold into microfluidic cardiovascular models and discuss how these methods have been adapted to effectively model disease processes not captured with traditional cell culture or animal disease models.

Of particular interest in cardiovascular physiology and pathophysiology is the dynamic remodeling of the ECM by resident cells in response to mechanical load, physiological stress, and circulating biochemical signals (Hynes, 2007; Humphrey, 2008). This remodeling is necessary to account for and adapt to changes in cardiac output and vascular resistance associated with aging, chronic disease, and/or wound healing, among other processes (Bonnans et al., 2014). Importantly, dysfunctional remodeling of the ECM is not only correlated with, but often is the underlying cause of various cardiovascular diseases. For example, collagen-rich fibrosis of the myocardium occurs in dilated and hypertrophic cardiomyopathy (Kapelko, 2001; Louzao-Martinez et al., 2016), and the loss of elastic fibers, aberrant collagen and elastin deposition, and increased vascular ECM stiffness are characteristics of hypertension, arterial aging and arterial calcification in response to inflammatory conditions (Arribas et al., 2006; Wagenseil and Mecham, 2012; Sun, 2015). Beyond its role in shaping and maintaining cardiovascular tissue architecture and mechanics, ECM also plays an important role in regulating growth factor transport and signaling, and dysfunctional ECM remodeling can exacerbate pathogenic cell signaling, contributing to the development of aortic aneurysm in rare diseases such as Marfan and Meester-Loeys syndrome (Kolb et al., 2001; Sakai et al., 2016; Meester et al., 2017; Thomson et al., 2019). Therefore, the development of cardiovascular disease models is not only dependent on the environment and mechanics at the time of cell seeding, but also on the ability of cells to remodel the ECM in response to biochemical and mechanical stimuli. This requirement for homeostatic remodeling of the ECM motivates the use of soft 3D substrates within microfluidic devices, which presents design and fabrication challenges as conventional processes for fabricating microfluidics result in stiff, planar surfaces (Griffith et al., 2020).

**Figure 2:**
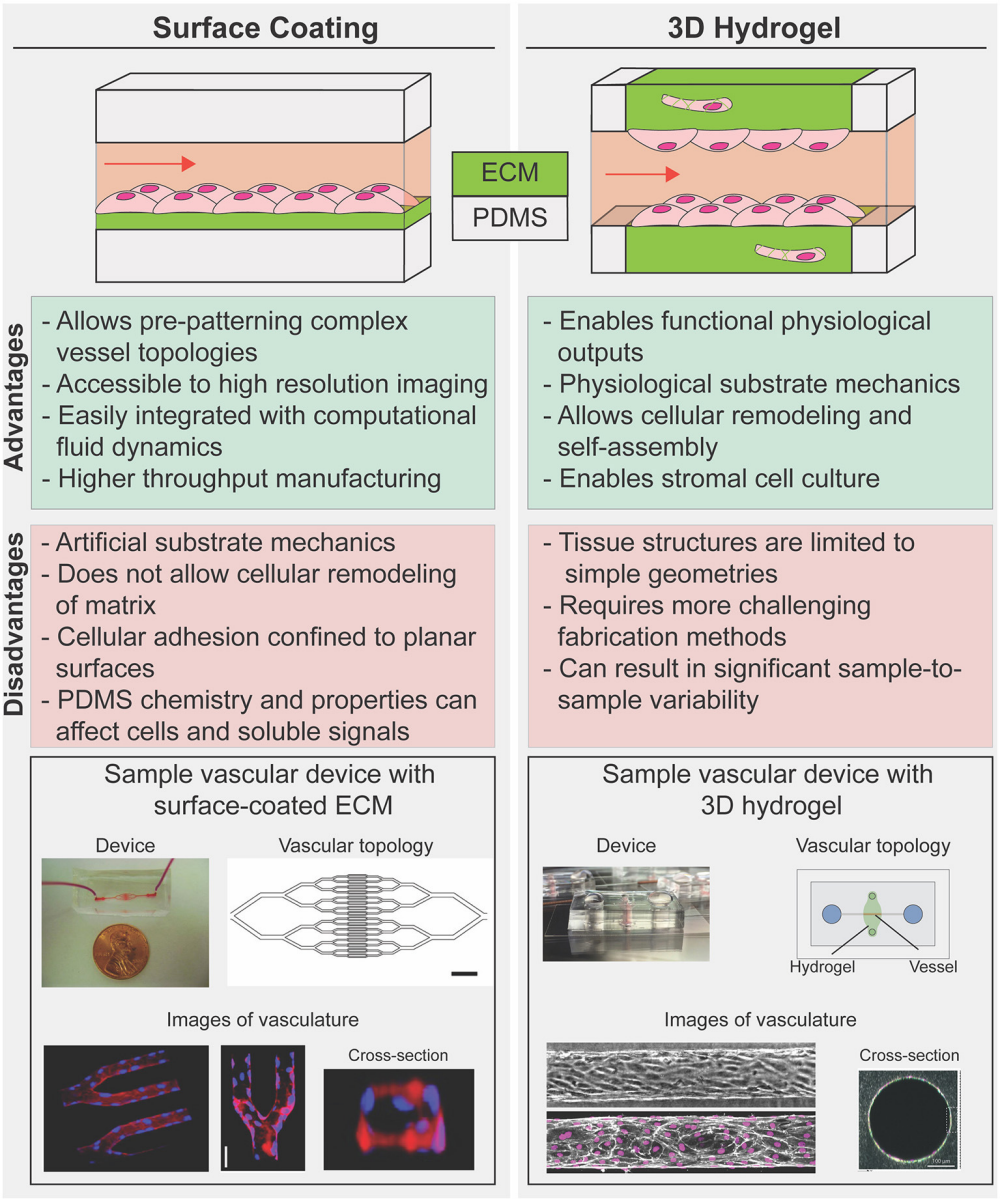
Strategies to reconstruct cells-ECM microenvironments in microfluidic disease models. 2D coated surfaces (left) and 3D hydrogels (right) each provide unique experimental advantages and limitations. Surface coated PDMS membrane is commonly used as substrate in platforms that have been used to investigate the effect of cyclic stress on cellular function or those that require complex geometry (bottom, left). 3D hydrogel systems are often limited to linear geometries but provide an optimal mechanical cellular microenvironment to study how spatial and mechanical cues in 3D influence vascular dynamics in diseases while allowing for cellular co-culture and hydrogel remodeling (bottom, right). [bottom, left: Images republished with permission of American Society for Clinical Investigation from Tsai et al. (2011); permissions conveyed through Copyright Clearance Center Inc. Bottom, right; Top: reproduced from Polacheck et al. (2019); Bottom: Reproduced from Wang et al. (2020) with permission from the Royal Society of Chemistry].

Various strategies have emerged for recapitulating native ECM in microfluidic disease models, with varying degrees of experimental difficulty and physiologic relevance. Generally, these approaches can be divided into two broad strategies: (1) surface coatings—reconstituted or synthetic ECM bound to a rigid structural material such as PDMS (Zhou et al., 2010); and (2) 3D hydrogels—highly hydrated 3D scaffolds that are comprised of networks of cross-linked polymer chains (Figure 2; Drury and Mooney, 2003). While 3D hydrogels allow for cellular remodeling of ECM composition and structure, inclusion of hydrogels within microfluidics constrains device design and presents experimental and technical challenges associated with applying fluid pressures within and across compliant porous materials (Polacheck et al., 2013; Akbari et al., 2017; Park et al., 2019; Griffith et al., 2020; Low et al., 2020). Therefore, microfluidic devices fabricated with surface coatings are often more sophisticated in terms of device structure and enable application of more physiologically relevant biophysical cues such as cyclic stretch and high fluid pressures and shear stresses, while those fabricated to include 3D hydrogels sacrifice device complexity for more physiologic culture substrates. For further information on hydrogel chemistry for tissue engineering and on microfluidic device design for modeling the vasculature, we refer the reader to relevant comprehensive reviews (Drury and Mooney, 2003; Wong et al., 2012; Huber et al., 2018). Here, we focus on approaches developed specifically for cardiovascular disease modeling and we compare and contrast surface coatings and 3D hydrogels to highlight benefits and drawbacks to each approach. We conclude with a brief discussion on how recent advances in patterning 3D hydrogels might lead to improvements in cardiovascular disease modeling.

### ECM Surface Coatings

Various strategies have been developed to coat polydimethylsiloxane (PDMS, the material conventionally used to fabricate microfluidic devices) or glass with ECM-like components from passive adsorption of purified matrix proteins such as collagen (Parenteau-Bareil et al., 2010; Gorgieva and Kokol, 2011) to covalent crosslinking of synthetic ECM presenting specific adhesion ligands (Zhou et al., 2010; Chen et al., 2018). These surface coatings often consist of single or combinations of purified ECM proteins such as collagen, fibronectin, or laminin, which allow for cell adhesion and while these ECM proteins are relevant to cardiovascular ECM composition, they do not completely recapitulate the complexity and tissue-specific combinations of *in vivo* ECM microenvironment. In general, surface coatings impose few restrictions on the device design and fabrication process and thus allow for cell culture in devices with complex geometry. For example, Tsai et al. coated ~30 μm branching PDMS channels with fibronectin as a substrate for endothelial cells to enable perfusion of human blood to assess the efficacy of vascular occlusion treatment in sickle cell disease and hemolytic uremic syndrome (Figure 2; Tsai et al., 2011). An added benefit of surface coating is the ability to culture and image cells in close proximity to a microscope objective to enable high resolution imaging. The authors leveraged this optical advantage to measure occlusion and blood flow velocity within patterned channels to determine whether hydroxyurea, a common treatment for sickle cell disease, impacts blood flow, and occlusion formation. The authors induced hemolytic uremic syndrome-like vascular injury and thrombosis and then tried to recover vessel health through treatment with eptifibatide, a drug that disrupts platelet aggregation. Through examination of clotting within the device, the authors found decreased thrombi formation after treatment, indicating that eptifibatide could be an effective treatment for hemolytic uremic syndrome. Similar approaches have been used extensively to study the role of hemodynamics in platelet activation and thrombosis, as recently reviewed (Zhu et al., 2015; Zhang and Neelamegham, 2017; Herbig et al., 2018).

PDMS affords certain advantages and disadvantages as a culture substrate in cardiovascular disease modeling. While PDMS cannot be remodeled by cells and is generally much stiffer than microvascular ECM (Polacheck and Chen, 2016), its elastic modulus can be tuned to values ranging from 0.1 to 2,700 kPa, which can better mimic physiological stiffness for larger vessels and the myocardium (Brown et al., 2005; Lv et al., 2017). Further, the high toughness and elasticity allow reversible and cyclic strain to be generated in PDMS under driving pressures easily achieved with standard microfluidics. In a highly influential platform technology, Huh et al. coated porous PDMS membranes with either collagen or fibronectin coating as a substrate for endothelial cells and alveolar epithelial cells, which could mimic the cyclic breathing motion of the alveolus (Huh et al., 2010). This system has since been modified to study many cardiovascular diseases including Hutchinson-Guilford progeria syndrome (Ribas et al., 2017) and cardiac fibrosis (Ugolini et al., 2016). For example, Zheng et al. leveraged this device design to simultaneously apply fluid shear stress and cyclic stretch to endothelial cells to recapitulate the mechanical conditions associated with early atherosclerosis (Zheng et al., 2016). They found an increase in reactive oxygen species formation in response to either abnormal fluid shear or cyclic stress, and the effect was more pronounced when cells were subjected to both stresses. They further evaluated endothelial cell response to inflammatory and hyperglycemic conditions and demonstrated increased cell death in endothelial cells cultured in device as compared to 2D cultured cells. Further, treatment of endothelial cells cultured in device under physiological flow and cyclic stress with probucol, a drug pulled from the market due to off target cardiovascular effects, uncovered cardiotoxicity not seen in a traditional cell culture model. They further showed that their device presented similar results to mouse model after treatment with antioxidant functionalized nanoparticles for hyperglycemia. Together these results demonstrate that despite the superphysiological stiffness, lack of cellular remodeling and relevant ECM complexity, microfluidic devices synthesized with ECM-coated PDMS or glass have provided novel insight into cardiovascular disease mechanisms and drug efficacy through their ability to recapitulate mechanical aspects of the microenvironment.

### 3D Hydrogels

To address the limitations of surface coated PDMS and glass, approaches have been developed to incorporate 3D hydrogels within microfluidic devices. In these systems, the PDMS microfluidic device serves as an initial template and gasket for fluidic access to a dynamic cell-laden hydrogel to mimic cardiac or vascular tissue. There has been considerable interest and effort in patterning hollow channels through the hydrogel to mimic microvascular architecture while enabling perfusion and the establishment of solute and fluid pressure gradients (Figure 2; Song and Munn, 2011; Nguyen et al., 2013; Verbridge et al., 2013; Polacheck et al., 2017; Kutys et al., 2020). These systems are particularly useful for studying the effect on disease presentation of changes in surrounding ECM composition and structure. For instance, the use of degradable porous materials as bulk hydrogels in microfluidic platforms have enabled investigations into the role of substrate stiffness (Bordeleau et al., 2017), chemical gradients (Kim et al., 2013; Nguyen et al., 2013), and fluid flow (Shin et al., 2011; Song and Munn, 2011) in directing endothelial sprouting and angiogenesis—a process that has been associated with atherosclerosis and ischemic heart disease (Khurana et al., 2005). Using a dextran hydrogel, Trappmann et al. investigated the material properties that impact angiogenesis and demonstrated that the degree of ECM degradability influences endothelial cell migration during angiogenesis (Trappmann et al., 2017). Despite the lack of a biologically relevant ECM microenvironment, synthetic hydrogels can be functionalized with integrin binding sequences and cell degradable motifs which allow mimic and study of specific ECM properties such as binding capacity and matrix degradability in development and disease. Specifically, the authors modulated the matrix degradation rate by cross-linking protease sensitive sequences with different protease binding affinities to the hydrogel, and showed that depending on matrix degradability, endothelial cells could migrate as single cells or collectively as a multicellular unit. These results suggest that the angiogenic response to cytokine gradients can vary according to the composition of vascular matrix and local concentration of matrix metalloproteinases. Notably, these observations were enabled by the ability to establish molecular gradients across soft 3D ECM with the integration of high-resolution 3D imaging in microfluidic devices and would not have been captured in traditional 2D cell culture models.

Blood and lymphatic microvessels synthesized in microfluidic devices that incorporate 3D hydrogels have been shown to be effective models for investigating vascular barrier function, as transport between the luminal and surrounding hydrogel compartments can be visualized with high spatiotemporal resolution (Chrobak et al., 2006; Wong et al., 2012; Polacheck et al., 2017). These 3D vascularized models are typically encapsulated in collagen hydrogel, which mimics the multiscale organization of vascular ECM, and allows study of the dynamics of cell-ECM interactions and ECM remodeling in a physiologically relevant setting. Barrier function as measured by vascular permeability is a common indicator of vessel health and is dysregulated in many diseases, including atherosclerosis, cancer, lymphatic malformations, neurological disorders, and chronic inflammation (Bischel et al., 2013; Buchanan et al., 2014; McCurley et al., 2017; Partyka et al., 2017; Polacheck et al., 2019), and tissue-specific 3D vascularized models have been developed to explore the role of vascular permeability in disease progression. For example, Lugo-Cintron et al. developed a microfluidic platform to investigate the role of desmoplasia in modulating lymphatic permeability during breast cancer metastatic progression. The authors demonstrated decreased lymphatic barrier function with increased collagen density, which mimics desmoplasia and associated increased collagen deposition and tissue stiffening. In particular, they found that secretion of pro-inflammatory cytokine, IL-6 increases in a collagen density-dependent manner. They further demonstrated that targeted blocking of IL-6 receptor is sufficient to mitigate vessel leakiness. Similarly, IL-6 was found to mediate blood vascular dysfunction in a model of early breast cancer adapted to include a polarized epithelial duct (Kutys et al., 2020). In a similar bulk hydrogel system, Silvestri et al. developed a tumor organoid-vessel co-culture model to study the process of vascular recruitment and tumor intravasation. The model is designed to be compatible with live cell imaging and allow for real time visualization of tumor-vessel interaction within a physiological relevant ECM microenvironment. Using this platform, they identified multiple cellular mechanisms where tumor cells could interact, displace, and disrupt vascular function (Silvestri et al., 2020). Importantly, these results highlight the utility of the advanced imaging methods that come with using microfluidic platforms for studying cellular processes that are difficult to capture in animal disease models. Overall, these results underscore the importance of incorporating physiological relevant ECM protein into the microenvironment of vascular disease models to provide functional readouts of vasculature in relation to disease progression.

A number of microscale and microfluidic models of functional, contractile cardiac tissue have been made utilizing 3D hydrogels. We direct the reader to recent comprehensive reviews for the development of cardiac-on-chip technology, cardiac microtissues, and microfluidic models and focus our review on platforms that have been used for disease modeling (Ribas et al., 2016; Ronaldson-Bouchard and Vunjak-Novakovic, 2018; Kitsara et al., 2019). Broadly, by encapsulating cardiac cells within 3D hydrogels, these systems allow for the study of cardiac tissue mechanics with (Xiao et al., 2014; Marsano et al., 2016) or without active mechanical stimulation (Legant et al., 2009; Boudou et al., 2012; Aung et al., 2016). These platforms are important tools for cardiac disease modeling as they can be made with iPS-derived cardiac cells (Thavandiran et al., 2013; Hinson et al., 2015; Lu et al., 2017; Ronaldson-Bouchard et al., 2018). While many of these approaches are not traditionally microfluidic in that they do not include enclosed microfabricated channels, they are fabricated with similar approaches and afford many of the benefits for disease modeling discussed in this review.

In general, the models referenced above are created by mixing cardiomyocytes and fibroblasts with a synthetic or natural hydrogel and depositing this mixture into a well with parallel, vertical microposts. Over time, these cells remodel the hydrogel into a dense microtissue that is anchored by the microposts, and micropost deflection can be used to measure the contractile forces generated by the tissue (Polacheck and Chen, 2016). Cardiac microtissues have been used to assess cardiac structure and function in response to patient mutations (Hinson et al., 2015), and increasingly to assess cardiotoxicity as a measure of drug safety. For example Lu et al. generated a 96-well array of cardiac microtissues from iPSC derived cardiomyocytes to assess how eight different drugs with clinically-observed cardiotoxicity affected beating rates and overall viability of the microtissues (Lu et al., 2017). Interestingly, their results showed that many of these drugs did not cause cell death at high doses but did result in lower overall beating rates, a measure of cardiac function. These results demonstrate that the functional measurements enabled by these microtissues provide improved clinical relevance for safety studies over traditional cardiomyocyte viability assays, highlighting the utility of microscale models for drug screening.

## Cardiovascular Tissue Morphology

The substantial remodeling of blood vessels and vascular networks during tissue growth and morphogenesis during development highlights the intimate and reciprocal relationship between cardiovascular structure and function. While there is some debate as to the relative roles of genetic prespecification vs. adaptation to mechanical load (Jones et al., 2006), results from developmental and animal disease models have made it increasingly clear that consideration of both genetics and morphology are critical in the study of cardiovascular disease, where: (a) genetic mutations can result in dysfunctional structural adaptation that increases morbidity and mortality, for example, in vascular Ehlers-Danlos syndrome where defective collagen production leads to weakened blood vessels, aneurysm formation, and rupture (Bergqvist et al., 2013; Malfait et al., 2020); and (b) tissue morphology can exacerbate disease, for example, curvature and branches in the descending aorta can lead to local hemodynamic recirculation and atherosclerosis (Chiu and Chien, 2011). Through integration of genetic models of disease with approaches for patterning 2D and 3D ECM, as discussed above, microfluidic models have enabled novel insight into the structure-function relationships that underlie cardiovascular disease progression. Due in part to the complex multiscale structure of the vasculature and to the role of hemodynamics in disease progression, much more effort has been devoted to the development of microfluidics for understanding the role of morphology in vascular disease modeling. Thus, we refer the reader to recent reviews on cardiac disease modeling (Mathur et al., 2016; Ribas et al., 2016; Ronaldson-Bouchard and Vunjak-Novakovic, 2018) and focus the subsequent section on models of the vasculature.

**Figure 3:**
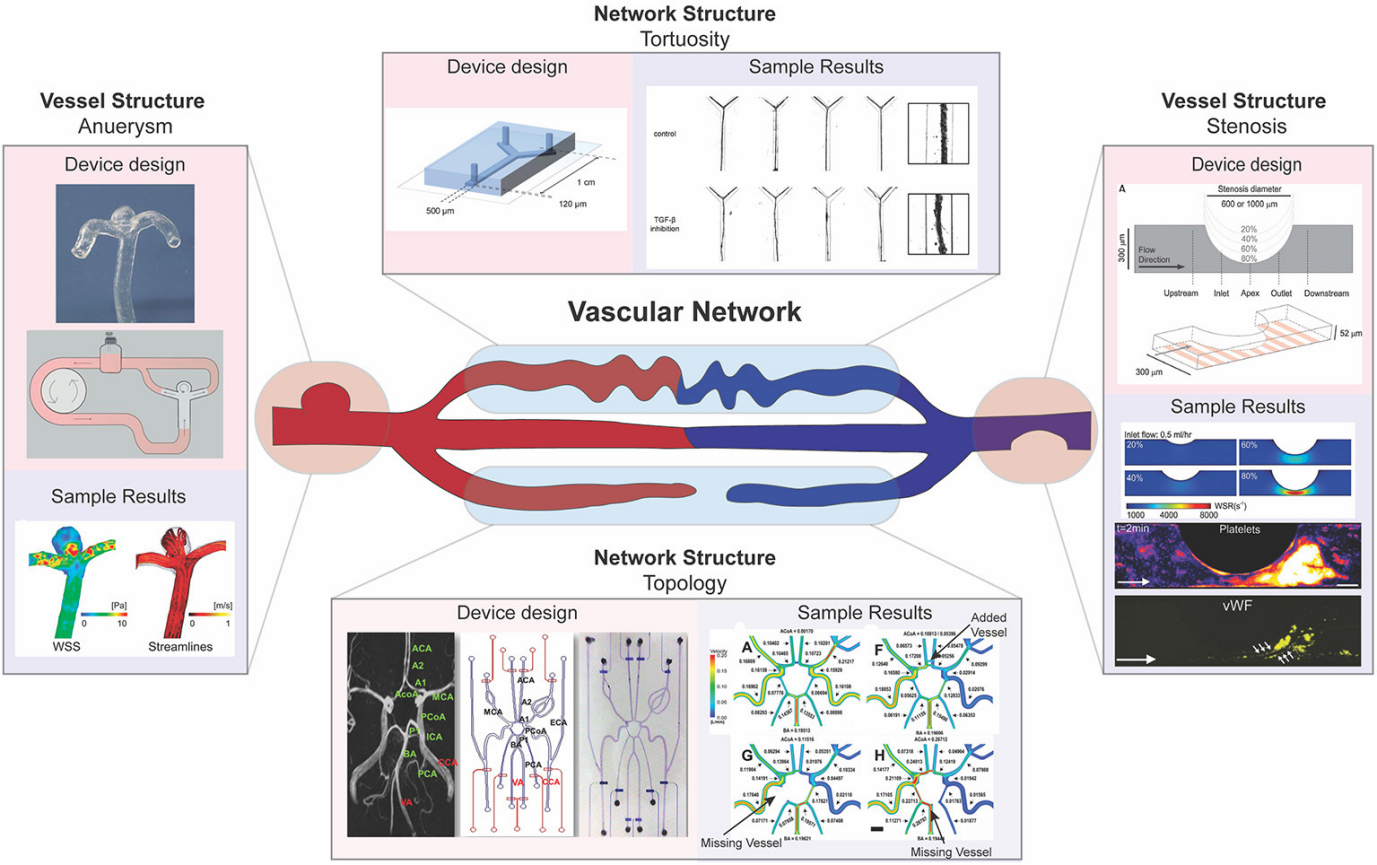
Multi-scale modeling of pathophysiologic vascular morphology associated with vascular diseases. Microfluidic models have been developed to model pathologic alterations in vascular morphology at the vessel and network levels. Examples of investigations into focal vessel pathologies include microfluidics designed to model aneurysm (left) and stenosis (right). Examples of investigations into network pathologies include microfluidics designed to model high vessel tortuosity (top) and developmental pathologies in the circle of Willis (bottom). Functional outputs highlighted include computational fluid dynamic studies using microfluidic system morphology (left, right, bottom), quantitative and qualitative validation of vessel size and shape (top), and biochemical analysis to assess adhesion (right). [left: images reproduced from Kaneko et al. (2018) with permission from BMJ Publishing Group Ltd. Right: images reproduced from Westein et al. (2013). Top: Images republished with permission of Royal Society of Chemistry from van der Meer et al. (2013); permissions conveyed through Copyright Clearance Center Inc. Bottom: images reprinted from Nam et al. (2015), Copyright 2015, with permission from Elsevier].

Broadly, pathologic vascular morphology manifests at the vessel level, including abnormal vessel length, tortuosity, or diameter, or at the network level, including abnormal vascular density and branching (Figure 3; Han, 2012; Xu et al., 2017). The location and dimensions of these abnormalities vary among patients, presenting challenges in the use of animal models to investigate clinical heterogeneity. A key benefit of microfluidics is the ability to develop customized cardiovascular models whose dimensions and complexity can be modulated to recapitulate patient-specific morphologies (Piccinelli et al., 2009; Nam et al., 2015; Costa et al., 2017; Kaneko et al., 2018). Such approaches, developed using micro- and nanoscale patterning and printing, have been effective in elucidating patient specific abnormalities, and the details of model fabrication are reviewed previously by others (McDonald et al., 2000; Ho et al., 2015; Amin et al., 2016; Griffith et al., 2020). Furthermore, the vascular system is subjected to fluid flow and shear stress, variables that can be drastically altered by deviations from normal in vessel morphology (Han, 2012). Using the fabrication methods mentioned above, microfluidics can be used to model severe changes in vessel morphology that correspond to cardiovascular disease and assay the impact of these changes on fluid flow and shear stress profiles. Here, we review implementation of these microfluidic approaches that have been used to understand the mechanisms underlying how focal and global changes in vessel morphology contribute to vascular disease.

Aneurysms are a focal change in vessel structure that increase in prevalence with age and are associated with a variety of disorders such as diabetes, Marfan syndrome, and vascular Ehlers-Danlos syndrome (Muluk et al., 1994; Bergqvist et al., 2013; Raffort et al., 2018). They occur when a blood vessel wall weakens at a specific point and begins to bulge, filling with blood and disturbing blood flow patterns. As fluid builds up in this newly formed bulge, the wall is weakened further, and changes in blood pressure and wall shear stress can eventually lead to rupture (Li and Kleinstreuer, 2005). The progression of aneurysms to dissection has been studied using computational fluid dynamic (CFD) modeling and has shown to be influenced by both aneurysm morphology and hemodynamics (Field, 2003; Xiang et al., 2011). However, using CFD with clinically obtained images alone does not allow for the impact of each of these factors—morphology and blood flow—to be individually analyzed. Reece et al. created a two-dimensional microfluidic model that can be used in tandem with particle image velocimetry to investigate how vessel structure changes due to aneurysm alter fluid flow and wall shear stress to trigger clotting within aneurysms (Reece et al., 2015). Their system allows for the visualization of the flow of beads or cells and thus provides a method for understanding how blood constituents flow differently through the aneurysm and initiate clotting in patients. Platforms created by Mannino et al. and Kaneko et al. built upon this two-dimensional flow model by recapitulating aneurysms using a three-dimensional microfluidic model with hydrogels, the first using more traditional techniques and the latter using patient MRI scans to inform 3D prints to generate their device's structure during fabrication (Mannino et al., 2015; Kaneko et al., 2018). Both groups used their microfluidic models to confirm previous CFD results based on clinical images and show wall shear stress is lower within the aneurysm sac (Figure 3). Kaneko et al. further showed that endothelial cells in the aneurysm sac differed in shape from those in the vessel lumen when exposed to fluid flow (Kaneko et al., 2018). By examining cell morphologies at different spots within the vessel, these results give indication of how changes on a cellular level could lead to further weakening and rupture of aneurysms. Collectively, these studies showcase how microfluidic models allow the examination of both the hemodynamics present in the vasculature, such as shear stress and flow profiles, and cellular phenotypes present within aneurysm, a vascular condition with a rather non-uniform morphological presentation.

Stenosis, or abnormal narrowing of blood vessels, is caused by a buildup of plaque in arteries or veins and is the major disease characteristic of atherosclerosis and thrombosis. Over time, these plaques rupture into precipitating thrombi that obstruct blood flow, and can result in stroke or myocardial infarction (Libby et al., 2019). Westein et al. observed that platelets tend to aggregate downstream of a stenosed mouse artery and developed a microfluidic model to identify hemodynamic changes that could contribute to downstream platelet aggregation. The authors fabricated a microfluidic device that recapitulated the stenosed structure and examined how factors such as wall shear stress, stenosis geometry, and activity of coagulation factors such as von Willebrand factor (vWF) changed along the stenosed structure (Figure 3). Their results showed that increased wall shear stresses at the stenosed region correlates with vWF activation and an increased capacity of platelet aggregation downstream to the site of stenosis, highlighting how microfluidic devices can be used to probe different aspects of disease presentation (Westein et al., 2013). Costa et al. took a three-dimensional approach to studying how platelet aggregation is affected by stenosis and used patient CT scans to generate 3D printed, endothelialized, stenosed vessels within a microfluidic chip. The authors fabricated cylindrical vessels with decreased diameter resembling points of stenosis and assessed thrombus formation by visualizing platelet accumulation after perfusion (Costa et al., 2017). Their results showed that changes in blood flow dynamics alone, as assessed by their microfluidic device, could not explain the location of thrombosis, suggesting that there is more to be discovered, and highlighting how the incorporation of patient-informed structure into microfluidic models has the potential to unveil mechanisms unable to be identified using traditional methods.

In addition to local changes in vessel structure, microfluidics have been developed to investigate the role of vascular network structure and remodeling in disease. For example, morphological variation, including absent vessels, within the circle of Willis (a complex network structure in the cerebrum) has been linked to cerebral aneurysm formation due to alteration in blood flow within this network (Nam et al., 2015). To study the relationship between wall shear stress resulting from this altered blood flow, Nam et al. fabricated a microfluidic device that recapitulates normal and disease state circle of Willis structures (Figure 3). The authors measured flow rates in the different morphological conditions and used CFD studies to show that higher shear stress correlates with aneurysm formation location in each of these conditions (Nam et al., 2015). Additionally, extreme deviation from straight vessel morphology has been implicated in various cardiovascular diseases, including atherosclerosis and ischemic heart disease (Han, 2012). As such, increased blood vessel tortuosity can be a measure of disease presentation, such as in hereditary hemorrhagic telangiectasia (HHT), a disease associated with disrupted TGF-ß signaling (Sawabe et al., 2001; Jaskolka et al., 2004). To investigate how TGF-ß signaling might impact tortuosity, van der Meer et al. fabricated a microfluidic device that contained endothelial cells and pericytes within a collagen hydrogel (Figure 3). By using a collagen hydrogel, these cells are able to remodel the hydrogel to form a self-organized vascular network, which allows probing of signaling pathways involved in vascular network formation (van der Meer et al., 2013). The authors observed that inhibition of TGF-ß signaling leads to increased vessel tortuosity, which implies that disrupted TGF-ß signaling could exacerbate vessel tortuosity, potentially leading to the development of twisted vascular network structure occurs in HHT. These results highlight the utility of microfluidic models to recapitulate tissue level structure and morphology in investigating disease presentation, which is extremely important in vascular diseases where morphology can impact fluid dynamics, and in turn vascular health.

## Patient-Derived Cells and Genetic Mutations

Due to the small sample sizes and compatibility with primary and patient-derived cells, microfluidic devices also allow the study of genetic disorders under physiologically relevant conditions. These systems are particularly useful for vascular disorders with distinct functional outputs that cannot be recapitulated using traditional *in-vitro* cell culture methods. Furthermore, genetic disorders are commonly caused by a wide diversity of driver gene mutations, which makes modeling the genetic landscape of human disease costly and challenging with animal models. The development of efficient gene editing, stem cell differentiation, and the increasing availability of patient-specific induced pluripotent stem cell (iPSC) lines have enabled the creation of genetically and physiologically relevant organ-on-chip systems for *in-vitro* disease modeling and drug testing. Here, we explore recent progress in genetic cardiovascular disease models, with an emphasis on the integration of patient iPSC derived cardiovascular cells in heart-on-chip and vascular-on-chip platforms for modeling pathologies in genetic disorders, including Barth syndrome, dilated cardiomyopathy, and Hutchinson-Gilford progeria syndrome. For a comprehensive review on the development of microfluidic devices for studying cardiovascular biology and drug development, see Ribas et al. (2016) and Ronaldson-Bouchard and Vunjak-Novakovic (2018).

**Figure 4:**
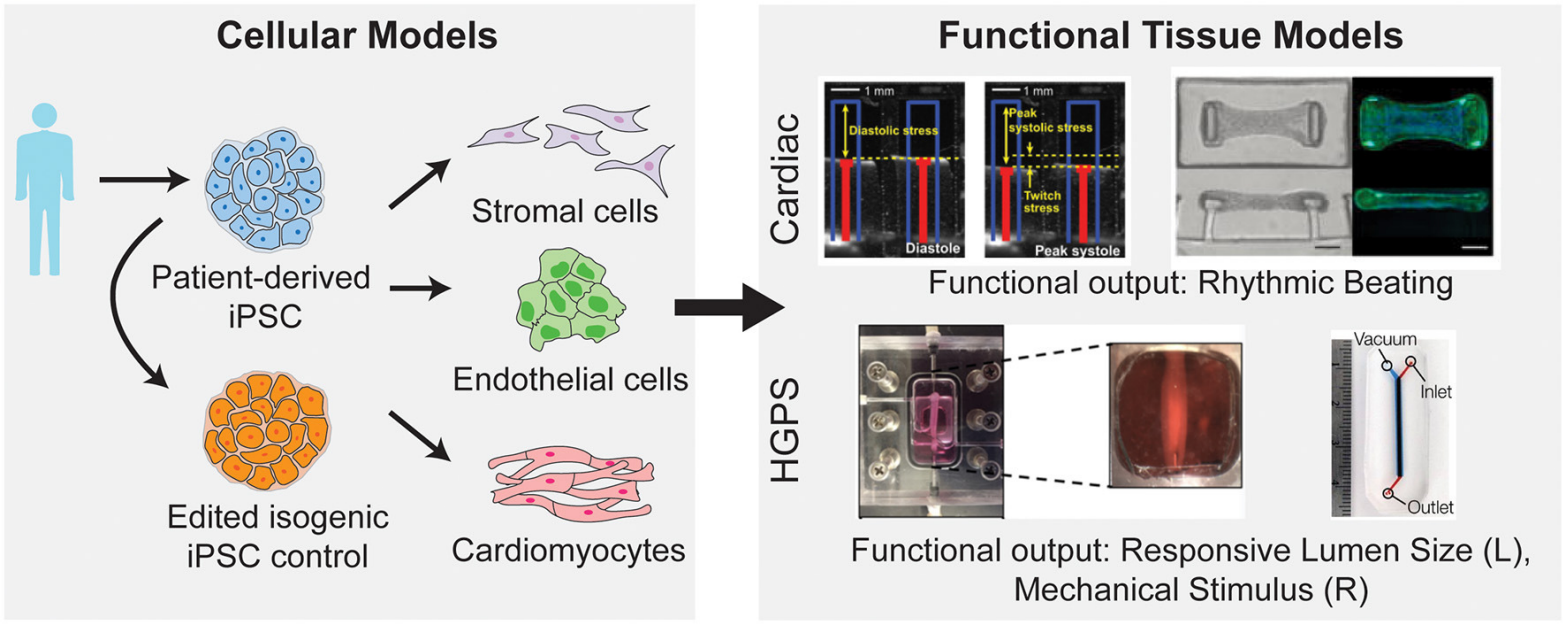
Integration of patient derived iPSC with microfluidic models to study cardiovascular diseases. iPSCs can be generated from patient cells and subsequently differentiated into a variety of vascular cell types. Further these iPSCs can be genetically mutated to create an isogenic control cell line to preserve the genetic background of the patient but erase the mutation of interest (left). After differentiation into vascular cells, these cells can be used in microfluidic disease models of both cardiac and vascular diseases (right). By utilizing microfluidic systems, the disease model can incorporate functional outputs as indicated [top, right; image on the left reprinted by permission from Springer Nature Customer Service Centre GmbH: Springer Nature, Wang et al. (2014), Copyright 2014. Image on the right from Hinson et al. (2015). Reprinted with permission from AAAS. Bottom, right; image on the left reproduced from Atchison et al. (CC by 4.0: http://creativecommons.org/licenses/by/4.0/). Image on the right reprinted with permission from John Wiley and Sons from Ribas et al. (2017), Copyright 2017 WILEY-VCH Verlag GmbH & Co. KGaA, Weinheim].

Traditionally, cardiovascular microfluidic models were limited, as cardiovascular cells are difficult to acquire from patients. The generation of iPSCs and their subsequent differentiation into vascular cells (Figure 4) allowed for increasing adoption of cardiovascular microfluidics, as these approaches overcame the previous lack of human vascular cells and allowed for modeling of patient-specific genetic mutations. For example, Wang et al. developed a heart-on-chip platform to model cardiomyopathy in Barth Syndrome using iPSCs (Wang et al., 2014). Barth Syndrome (BTHS) is a rare metabolic disorder caused by recessive loss-of-function mutations in the tafazzin (TAZ) gene that results in dilated cardiomyopathy, skeletal myopathy, neutropenia, and short stature (Barth et al., 1983). TAZ catalyzes acylation of monolysocardiolipin into cardiolipin, a phospholipid that is essential for electron transport chain and ATP synthase function (Chicco and Sparagna, 2007; Houtkooper et al., 2009). In a recent study conducted by Wang et al., iPSCs derived from BTHS patients were differentiated into cardiomyocytes and were shown to have impaired cardiolipin acylation, increased reactive oxygen species production and other metabolic abnormalities associated with BTHS (Wang et al., 2014). The authors further employed a muscular thin film tissue construct (Grosberg et al., 2011) to demonstrate contractile dysfunction in BTHS (Figure 4). Cardiomyocytes on thin elastomeric membrane aligned to micropatterned lines and organized into strips of anisotropic muscle tissues. The muscular thin film bends and deflects the membrane during contraction which serves as a functional readout for contractile force. Using this model, they showed that BTHS-derived cardiomyocytes exhibit compromised contractile performance as compared to control cardiomyocytes. They further employed this platform to evaluate potential therapies for BTHS and demonstrated that multiple strategies—reintroducing tafazzin with modified RNA, buffering mitochondrial reactive oxygen species with antioxidant, or providing cells with linoleic acid, an alternative precursor to cardiolipin, can be used to rescue the disease phenotype. The results from this study not only provide novel mechanistic insights into BTHS pathogenesis but also demonstrate the utility of patient-derived iPSC cardiomyocytes and heart-on-chip technology for therapeutic discovery.

Another application for human iPSC-based heart-on-chip platform is the mechanistic investigation of incomplete penetrance in dilated cardiomyopathy caused by titin truncating mutations (Hinson et al., 2015). Titin, the largest known human protein, is known to function structurally as a scaffolding protein for sarcomere assembly and mechanically as a molecular spring that provides elasticity to the muscle (Granzier and Labeit, 2004; LeWinter and Granzier, 2010). The protein is composed of four structural domains: the N-terminal Z-line domain that is anchored to the sarcomeric Z-disk, the distensible I-band region that is responsible for muscle elasticity, the thick filament binding A-band region that functions as a scaffold for filament assembly, and the C-terminal M band region with a kinase domain. In a landmark multi-cohort study of patients with dilated cardiomyopathy, Herman et al. showed that mutations leading to truncations in titin accounts for up to 25 and 18% of familial and sporadic dilated cardiomyopathy cases, respectively (Herman et al., 2012). The truncation variants found in dilated cardiomyopathy cohort clusters around the A-band region. Interestingly, truncating variants located in the I-band region were found in both patient and healthy population, suggesting that truncations located in I-band are better tolerated than truncations in A-band region (Herman et al., 2012; Roberts et al., 2015). Using patient-derived iPSC cardiomyocytes and gene-edited iPSC cardiomyocytes, Hinson et al. investigated the penetrance of titin truncation variants (TTNtv) and missense variants within the A-band and I-band regions (Hinson et al., 2015). The pathophysiology of each variant was evaluated using cardiomyocytes contractility assays, both in 2D single cell and 3D engineered cardiac microtissues (Figure 4). While difference in contractile performance was undetectable in individual cardiomyocytes, both A-band and I-band TTNtv lines displayed significantly lower contractile forces compared to control cardiac microtissues. Additionally, contractility defects were less pronounced in I-band TTNtv in comparison with A-band variants, indicating a reduced penetrance of the mutation. Using RNA sequencing, the authors demonstrated that the affected I-band exon can be alternatively spliced and excluded from mature titin transcript. They further suggested alternative exon splicing functions as the predominant mechanism for reduced penetrance of I-band TTNtvs. Collectively, the results of this study demonstrate a successful integration of iPSC, gene editing, transcription profiling with tissue engineering technology and provide a promising platform for studying genotype to phenotype relationships in genetic cardiac condition.

Stem-cell based vessel-on-chip systems have also been developed for modeling vascular dysfunction in rare genetic disorders. Progeria, or Hutchinson-Gilford progeria syndrome is an extremely rare, progressive genetic condition that causes premature aging. The disease is resulted from the buildup of progerin, a mutant nuclear lamin A protein and causes accelerated degenerative aging in multiple systems, including skin, musculoskeletal, and cardiovascular systems (Kudlow et al., 2007). In studies by Atchison et al., 3D arteriole-scale vessel-on-chip were generated with endothelial cells (Atchison et al., 2020) and smooth muscle cells (Atchison et al., 2017, 2020) derived from healthy and Progeria patient iPSCs (Figure 4). The tissue-engineered Progeria vessels were shown to have increased inflammatory cytokines and reduced vasoactivity as compared to control vessels. Using these systems, the authors demonstrated that vascular endothelium may play a specific role in initiating inflammatory response and the development of atherosclerosis in Progeria. João Ribas et al. further developed a Progeria-on-chip model to study the impact of pulsatile cyclic strain on pathophysiology of the disease (Ribas et al., 2017). iPSC derived smooth muscle cells were cultured on PDMS membrane and stimulated with continuous pulsatile stretch and relaxation (Figure 4). The progeria patient-derived smooth muscle cells showed increased levels of DNA damage, inflammatory markers and senescence markers in response to mechanical strain. Additionally, the authors showed that these phenotypes can be reduced with lovastatin or lonafarnib—a drug that was submitted for the first-ever Progeria treatment. These results point to the utility of patient stem cells in vessel-on-chip platforms to facilitate drug development for rare vascular diseases.

## Discussion

Over the past several decades, animal models for cardiovascular disease have enabled great advances in understanding of physiology and disease progression that have led to the development of compounds and therapies that have broadly changed the prognostic landscape for cardiovascular disease. Increasingly, microfluidic devices have contributed to the arsenal of experimental approaches available for understanding cardiovascular biology, and as the efficiency of drug development declines (Scannell et al., 2012), microfluidic approaches hold promise in addressing gaps in current preclinical models (Low et al., 2020). As the technology required for fabrication has been more widely adopted, these devices have moved beyond the proof of concept phase to enable the study of patient derived cells and mutations on disease progression at the tissue scale without sacrificing physiological relevance, thus bridging the gap between human and mouse physiology. Recent developments in 3D printing (Skylar-Scott et al., 2019) and in the development of advanced bioinks (Gladman et al., 2016) are further improving the spatiotemporal resolution with which cells and tissues can be patterned, and advances in organoid technology (Park et al., 2019) are enabling increasingly complex structures with cellular compositions more closely resembling native tissue. Integration of these technological advances with the ability to apply pressures and flows afforded by microfluidics will enable next-generation studies into the relationship between cardiovascular architecture and disease and open the possibility to develop these *in-vitro* systems into organ replacement or augmentation strategies.

While most recent work has focused on mutations that are conserved in diseased populations, the technology and methods developed to perform these studies will contribute to the development of individualized, patient-specific therapeutic interventions. As the cost and material requirements for gene sequencing continue to reduce, microfluidic cardiovascular disease models will be increasingly useful for understanding the functional consequences of individual and combinations of genetic mutations. Importantly, integration of disease models with information from electronic medical records will also allow study of comorbidities and patient history not well-captured by gene sequencing in cardiovascular disease progression. Such platforms and integrative approaches could also provide individualized preclinical safety and efficacy data to inform how a particular drug will impact a specific patient. Together, these advances will allow microfluidic cardiovascular disease models to study pathophysiological mechanisms and to screen for interventions in individual patients, and recent work supports the viability of this approach (Luna et al., 2020).

Currently, the development of microfluidic systems is intimately dependent on advances in animal models. Advances in understanding cardiovascular physiology inform the development of novel devices, and mechanistic insights into pathophysiology of cardiovascular disease enabled by microfluidics are largely validated with animal models. While this reciprocal relationship between animal models and microfluidics ostensibly ensures physiologic relevance of novel systems and assays, it presents a central obstacle in disease modeling and treatment development for diseases that are lacking translatable animal models. Furthermore, the lack of regulatory oversight on manufacturing standards and guidelines on device performance presents a bottleneck to clinical translation (Reyes-Hernandez et al., 2020). While recent companies have sought to commercialize specific chips, packaging and distribution of 3D hydrogels and cell-laden devices has yet to be achieved at the commercial scale. This requires individual labs to introduce ECM and cells into commercially available devices, which complicates standardization and contributes to a high degree of variability among research groups. As standard operating procedures and biomanufacturing continue to be developed to address these issues, challenges remain in regulatory interactions and strategy. Without standardized processes for determining the relevance and efficacy of microfluidic disease models, validation of new models requires recapitulation of key results in animal studies, which precludes development of microfluidic models for diseases in which animal models do not exist. While initiatives such as the Tissue Chip initiative led by the National Center for Advancing Translational Sciences in coordination with the Food and Drug Administration seek to address these challenges at a higher level, increasing coordination among investigators and laboratories developing the technology will improve adoption.

In summary, the integration of patient derived cells and mutations with microfluidic platforms has enabled novel insights into cardiovascular disease mechanisms that were not possible with standard *in-vitro* or animal disease models. Moving forward, continued advances in biochemistry and fabrication technology will enable these approaches to move beyond academic labs to significantly impact drug development for cardiovascular disease.

## Author Contributions

ED, WA, and WP drafted the manuscript and figures. WP and AH edited and provided comments on the manuscript and figures. All authors contributed to the article and approved the submitted version.

## Conflict of Interest

The authors declare that the research was conducted in the absence of any commercial or financial relationships that could be construed as a potential conflict of interest.
